# Dermal fibroblasts from patients with Parkinson’s disease have normal GCase activity and autophagy compared to patients with PD and GBA mutations

**DOI:** 10.12688/f1000research.12090.2

**Published:** 2018-02-09

**Authors:** Lucy M Collins, Janelle Drouin-Ouellet, Wei-Li Kuan, Timothy Cox, Roger A Barker

**Affiliations:** 1 Cambridge Stem Cell Institute, University of Cambridge , Cambridge , UK; 2 John Van Geest Centre for Brain Repair, University of Cambridge , Cambridge , UK; 3 Developmental and Regenerative Neurobiology, Wallenberg Neuroscience Center, Lund University, Lund, Sweden; 4 Department of Medicine, University of Cambridge, Cambridge, UK

**Keywords:** GBA mutations, Parkinson’s disease, Gaucher disease, fibroblasts, lysosome, autophagy

## Abstract

**Background: **Recently, the development of Parkinson’s disease (PD) has been linked to a number of genetic risk factors, of which the most common is glucocerebrosidase (GBA) mutations.

**Methods:** We investigated PD and Gaucher Disease (GD) patient derived skin fibroblasts using biochemistry assays.

**Results:** PD patient derived skin fibroblasts have normal glucocerebrosidase (GCase) activity, whilst patients with PD and GBA mutations have a selective deficit in GCase enzyme activity and impaired autophagic flux.

**Conclusions:** This data suggests that only PD patients with a GBA mutation have altered GCase activity and autophagy, which may explain their more rapid clinical progression.

## Introduction

Gaucher disease (GD) is an autosomal recessive condition that is caused by defective glucocerebrosidase (GCase) enzyme. GCase (GBA gene) normally functions to breakdown lipids in the lysosomes and mutations in GCase cause accumulation of its substrate, glucoceramide (GlcCer) ^1^. These mutations result in characteristic engorged macrophages, known as Gaucher macrophages (GMs) or ‘Gaucher cells’ ^2^. The GlcCer buildup in macrophages manifests as a multi-systems disorder, with signs such as hepatosplenomegaly, anaemia, thrombocytopenia, and bony impairments, reviewed by 3. GD is common in Ashkenazi Jewish populations, although the disorder is pan-ethnic ^4^. GD has been historically divided into three types, based on the severity of clinical features and neurological involvement ^3, 5^. Type I GD is classically defined as non-neuropathic, although neurological deficits have been described in some of these patients ^6^ including Parkinsonism ^7– 10^. Type II disease usually begins in infancy, with severe neurological involvement. Type III GD is an extended form of type II, also with neuropathic problems but patients live into adolescence and adulthood largely due to the development of enzyme replacement therapy (ERT) ^3, 5^.

It is now apparent that there is a strong genetic connection between Gaucher and Parkinson’s disease (PD). Studies have shown that in large multi-center patient cohorts, patients with PD have an increased incidence of carrying GBA mutations ^11– 14^, including 3% of sporadic PD patients and up to 15% in Ashkenazi Jewish populations with PD ^15^. Thus, we now know that heterozygote GBA mutations are the single commonest genetic risk factor for familial ^16^ and sporadic PD; leading to a more rapid progression of PD with an early onset dementia ^12– 14^.

The cellular pathology in lysosomal storage disorders (LSD) is centered around the misfolding of GCase and lysosomal dysfunction. Lysosomes have also been implicated in PD and several other neurodegenerative disorders including Alzheimer’s disease and Huntington’s disease ^17, 18^, suggesting that similar underlying defects in autophagy and lysosomal dysfunction may link the pathophysiology of PD to GD ^19^. The accumulation of substances in the lysosome impacts on their function and other intracellular pathways, resulting in secondary changes such as an impairment of autophagy ^20^. Lysosomal and autophagy impairments are apparent in cell line models, mouse models and in post-mortem tissue from PD patients. In PD mice models, components of lysosomal function are affected, causing a reduction in lysosomal number and accumulation of autophagosomes ^21^. Genetic mutations causing PD have also implicated the lysosome; these include ATPase Type 13A2 (ATP13A), which encodes a lysosomal ATPase, maintains lysosomal pH, and inhibits α-syn misfolding ^22^. α-synuclein aggregation in the lysosome, as seen in PD and LSD, may in turn accelerate its own aggregation.

In this study, we sought to investigate autophagic function and GCase enzyme function using cells derived from patients. We tested patients with PD with and without GBA mutations as well as individuals with GD. We sought to define the extent of reduced GCase enzyme activity in all cases, and how this relates to autophagic flux. The activity of three other housekeeping lysosomal enzymes was also looked at, to see the extent to which the enzyme defect was specific.

## Methods

### Patients and fibroblast cell lines

Fibroblasts were derived from dermal skin biopsies. In Table 1 the characteristics of the 13 patient derived and 4 healthy control fibroblast are summarised. Written informed consent was taken from each participant, and the ethics for this study was approved by the Cambridge Central Research Ethics Committee (REC09/H0311/88). HFL1 (ATCC-CCL-153) cells were obtained from the American Type Culture Collection (ATCC), expanded in standard fibroblast medium and used as controls. Skin biopsies were taken from patients with:

(i) Both GD and PD (homozygous, PD/GD), n=3
(ii) PD with one GBA mutation (heterozygous, PD GBA), n=3
(iii) PD with no GBA mutations, idiopathic PD (iPD), n=3
(iv) Healthy controls, n=4
(v) GD only (homozygous), with no PD (GD), n=4

**Table 1.** Clinical and demographic information for the patient fibroblast lines. WT = Wildtype, (?) = Unknown genotype.

Patient (ID, gender/age)	GBA Genotype	Clinical features	PD Family History
Parkinson’s disease/Gaucher disease PD/GD			
PD001 F/58	R463C/R463C	GD Type I/ PD	No
GD005 M/56	L444P/R463C	GD Type I/ PD	No
GD004 F/50	N370S/L444P	GD Type I/ PD	No
Parkinson’s disease GBA carriers (PD GBA)			
PD002 F/64	N370S/WT	PD	Yes
PD003 M/69	E326K/WT	PD	Yes
PD004 M/57	E326K/WT	PD	Yes
Parkinson’s disease normal GBA (iPD)			
PD005 F/68	WT/WT	PD	No
PD006 F/66	WT/WT	PD	Yes
PD007 M/65	WT/WT	PD	No
Healthy controls (Controls)			
C001 F/54	WT/WT	No neurological disease	No
C002 M/57	WT/WT	No neurological disease	No
C003 M/72	WT/WT	No neurological disease	No
C004 M/69	WT/WT	No neurological disease	No
Gaucher disease (GD)			
GD002 F/58	N370S/?	GD Type I	Yes
GD006 M/66	N370S/N370S	GD Type I	Yes
GD008 M/68	N370S/L444P	GD Type I	No
GD009 M/69	N370S/?	GD Type I	No

### Western blot experiments

To detect protein expression, samples obtained from the lysed cells were loaded on to a 4–12% SDS-PAGE gel (Invitrogen). Cells were harvested using either Radioimmunoprecipitation assay buffer (RIPA), containing 1.0% sodium dodecyl sulfate (SDS), 0.5% Sodium Deoxycholate, 0.1% Triton X 100, 150 mM NaCl, 50 mM Tris-HCl pH 8.0 and protease inhibitors, or NP40 (Nonidet P40) buffer containing 150 mM NaCl, 50 mM Tris-HCl pH 8.0, NP-40 1.0% and protease inhibitors. The samples were sonicated at 1 second intervals for 10 secs, centrifuged at 20,000 g for 20 minutes at 4°C and the supernatant was snap frozen and stored at -80°C. The total protein concentration was quantified by a BCA assay (Thermo Scientific). The gel was run in 3-(N-morpholino) propanesulfonic acid (MOPS) buffer at 100 V, 40mA for 1 hour. The proteins on the SDS gel were transferred to a polyvinylidene fluoride (PVDF) membrane at 30 V, 100 mA for 2 hours. Then the membrane was incubated with Ponceau red to detect the proteins before being blocked (room temperature (RT), 1 hr) with 5% w/v semi skimmed milk, 1% bovine serum albumin (BSA) PBS with tween (PBS-T 0.3% v/v) and incubated with primary antibodies GBA(GCase) rabbit polyclonal, sigma (G4046) 1:1000, LC3 rabbit polyclonal New England Biolabs (NEB) (2775S) 1:1000, and actin rabbit polyclonal Abcam ab8229 1:1000 overnight at 4°C. Following washes in PBS-T (3 X 5 min), horseradish peroxidase labeled secondary anti rabbit antibodies (Bio-Rad 1662408EDU) were applied at 1:10,000 dilution in 5% milk, 1% BSA (room temperature, 1 hr). Membranes were washed again with PBS-T and protein bands visualized by chemiluminescence (Thermo Pierce) estimating band molecular weights relative to standard protein markers in the range of 10–120 kDa (Novex® Sharp Pre-stained Protein Standard, Life Technologies). Protein bands were quantified by densitometry using Image J (Fiji) software.

### Immunocytochemistry for localisation of intracellular GBA proteins

The cellular localisation of the GBA protein in dermal fibroblasts was investigated using antibodies against GBA (Sigma, G4046, rabbit polyclonal 1:50) and Lamp1 (Abcam, ab25630, mouse monoclonal 1:50). Fibroblasts were grown to 50,000 cell/well confluency on 0.1% gelatin coated covers-slips in 24-well plates overnight in DMEM containing 10% FBS, and 1% penicillin/streptomycin (pen/strep) and fixed in 4% PFA for 30 minutes. Cells were washed twice with PBS, blocked in PBS, 10% FBS and 0.2% Triton and incubated for 1 hour at room temperature. After this the cells were then incubated overnight at 4°C with the relevant primary antibody (GBA (Sigma, G4046, rabbit polyclonal 1:50) and Lamp1 (Abcam, ab25630, mouse monoclonal 1:50)) in PBS, 0.2% Triton and 2% FBS. The cells were washed three times with PBS the following morning and incubated with the secondary antibodies (goat anti rabbit (green) and goat anti mouse (red) antibodies (Bio-Rad) were applied at 1:10,000 dilution in PBS, 0.2% Triton and 2% FBS in the dark for 2 hours at room temperature and washed with PBS three times for 5 minutes each and stained with Hoescht 1mg/ml in PBS for 15 minutes at room temperature in the dark. The cells were then washed with PBS 3 times for 5 minutes and imaged on a confocal microscope. In each well 10 fields of view were captured and this was repeated in triplicate.

### Bafilomycin assay

The dermal fibroblasts were seeded and grown at a density of 72,000 cells per well in 24-well plates overnight in DMEM with 10% FBS and 1% pen/strep. Bafilomycin A1 from Sigma-Aldrich (B1793) was used at a concentration of 0.1 mM and added to the cells for 2 hours, this concentration was derived from titrations of Bafilomycin A1 and 0.1 mM and found to be the concentration best tolerated by the cells. The cells were either treated with 1) Bafilomycin A1 alone, 2) Bafilomycin A1 and starvation medium (Hanks’ Balanced Salt Solution (HBBS) with 5% Sodium Bicarbonate) 3) starvation alone and 4) untreated cells as a control. The cells were incubated for 2 hours at 37°C and then washed twice with ice cold PBS. To harvest, the cells were incubated with prechilled NP-40 lysis buffer for 30 minutes with shaking at 4°C. The cells were then collected and spun at 20,000 g for 15 minutes, the supernatant was snap frozen and stored at -80°C until processed for western blotting analysis.

### Lysosomal activity assays

***Cell preparation.*** The enzyme activity assays were performed on fibroblasts harvested from 24-well plates. Cells were seeded at a density of 100,000 cells per well in DMEM containing 10% FBS and 1% pen/strep. After 24 hours the cells were harvested in ice-cold lysis buffer specific to each reaction described below. The lysate was spun for 20 minutes at 20,000 g at 4°C. The cell pellet was then resuspended in lysis buffer and sonicated for 1 minute, while keeping the sample on ice. A BCA assay (Thermo Fisher) was performed to quantify protein concentration.

### Glucocerebrosidase activity assay

For the GCase activity assay, cells were harvested in lysis buffer containing 30 mM Citrate Phosphate buffer pH 5.5, 0.65% Sodium Taurochlorate and 0.65% triton-X 100. Enzyme activity was measured using 4-Methylumbelliferyl (4MU) β-D-glucopyranoside, Sigma (M3633), as the substrate in running buffer containing 30 mM Citrate Phosphate buffer and 0.6% Sodium Taurochlorate pH 4.4 (to inhibit GCase 2 and 3). Each well in a 96 well black microplate, contained 20 μg (for each sample ~20 μl of protein and 30 μl of buffer) of sample protein, 75 μl running buffer and 25 μl of 15 mM substrate (which was made up immediately before the assay at 59.15 mg/ml in Dimethyl sulfoxide (DMSO) and diluted 1/10 in running buffer and kept in the dark). Each assay was repeated in triplicate for each cell sample. The sample alone was added in triplicate as a blank and also, the buffer with the substrate alone was added as a blank. The samples were incubated for 1 hour at 37°C and the reaction was stopped using 100 μl of 0.2 M Glycine pH 10.5. The fluorescence was read on the FLUOstar Omega plate reader at excitation 355 nm and emission 460 nm.

### Hexosaminidase activity assay

Total hexosaminidase activity was measured using fibroblasts harvested with lysis buffer containing 0.01 M Citrate phosphate buffer, pH 4.4, and 0.2 M Na _2_HPO _4_. A solution of 4 μg cell lysate (for each sample of ~10 μl of protein), 95 μl of 0.01 M Citrate Phosphate buffer and 20 μl of 2.5 mM 4-MU N-acetyl-b-D-glucosaminide, Sigma (M2133), in 0.01 M Citrate phosphate buffer was added to each well, the assay was performed in triplicate and blanks were included in lysate alone and a separate blank (buffer and substrate alone). The reaction ran for 20 minutes at 37°C and was stopped by adding 100 μl of 0.17 M Glycine pH 9.8 (made up from 2.5 g glycine and 3.6 g Na _2_CO _3_). The activity was measured on the FLUOstar Omega plate reader at excitation 360 nm and emission at 415 nm.

### Galactosidase activity assay

To measure α-galactosidase activity, 15 μg of cell lysate (each sample contained ~20 μl of protein and 30 μl of buffer), was incubated with 75 μl 0.2 M Citrate Phosphate buffer pH 4.4 and 20 μl of 10 mM 4-MUl α-D-galactopyranosidase, Sigma (M7633). This cell lysate solution was added to each well, the assay was performed in triplicate and blanks included lysate alone and a separate blank (buffer and substrate alone). The reaction was incubated for 30 minutes at 37°C and stopped by adding 100 μl of 0.17 M Glycine pH 9.8. The activity was measured on the FLUOstar Omega plate reader at excitation 360 nm and emission at 415 nm.

### Mannosidase activity assay

To measure mannosidase activity, 15 μg of cell lysate (for each sample ~20 μl of protein and 40 μl of buffer), was incubated with 50 μl of 0.2M Citrate Phosphate buffer pH 4.4 and 15 μl of 5 mM 4-MU α-D-mannopyranoside, Sigma (M3657). This cell lysate solution was added to each well, the assay was performed in triplicate and blanks included lysate alone and a separate blank (buffer and substrate alone). The reaction was incubated for 20 minutes at 37°C and stopped by adding 100 μl of 0.17M Glycine pH 9.8. The activity was measured on the FLUOstar Omega plate reader at excitation 360 nm and emission at 415 nm.

### Gold nanoparticle analysis

The fibroblasts were plated at a density of 50,000 cells/well on 0.1% gelatin coated coverslips and placed in a 24-well plate. The cells were grown over night in DMEM with 10% FBS and 1% pen/strep. The following day Gold nanoparticles were added to the cells (AuNPS). The cells were incubated with the AuNPS for 24 hours and then washed with cell culture medium to remove the AuNPS. After 16 hours the cells were fixed using 4% formaldehyde and imaged on a brightfield microscope.

### Statistical analysis

All analysis was performed using SPSS version 20.1 (IBM). Normality for all the variables was tested using a one-sample Kolmogorov–Smirnov tests. Variables which did not follow a normal distribution, were log transformed and retested for normality. Non-parametric variables were compared with a Mann-Whitney U-test or Kruskal Wallis test. Parametric variables were compared with a t-test or ANOVA. Bonferroni post analysis was applied. Each experiment was repeated a minimum of three times.

## Results

### GCase activity is specifically decreased in patient fibroblasts carrying a GBA mutation

**Figure 1.**
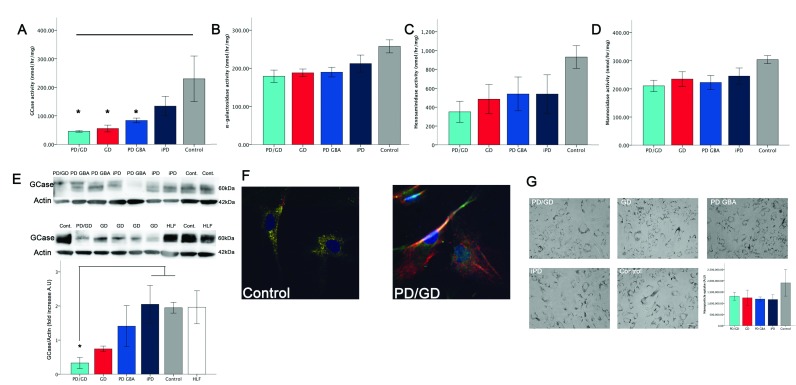
Lysosomal enzyme activity assays and Gold nanoparticle uptake assays. ( **A**) GCase activity was significantly lower in the PD GBA (83.76 ± 8.16) (p=0.023), GD (55.33 ± 11.88) (p=0.006) and PD/GD (45.56 ± 2.99) (p=0.013) compared to controls (229.67 ± 79.96). The iPD group was not significantly different compared to any other groups (134.20 ± 33.94) (p>0.05) ( **B**) α-galactosidase activity for PD (212.42 ± 22.30), PD GBA (189.81 ± 12.51), GD (188.10 ± 10.02), control (257.63 ± 17.13) and PD/GD (179.00 ± 16.25) (p>0.05). ( **C**) Hexosaminidase activity for PD GBA (540.10 ± 178.66), iPD (539.61 ± 203.33), GD (485.08 ± 153.99), control (931.33 ± 120.93) and PD/GD (350.65 ± 111.99) (p>0.05). ( **D**) Mannosidase activity for iPD (245.33 ± 28.76), PD GBA (222.67 ± 24.81), GD (234.83 ± 26.06), control (304.00 ± 14.00) and PD/GD (210.44 ± 19.92) (p>0.05). Enzyme activity measured by the FLUOstar Omega plate reader (Ex max = 360nm, Em max.= 415nm. ( **E**) Western blot of GCase protein levels. No reduction in GCase expression in GD (0.74 ± 0.08), PD GBA (1.41 ± 0.60) and the HLF (1.96 ± 0.48) (all p>0.05). PD/GD (0.33 ± 0.16) was significantly lower compared to control (1.95 ± 0.15) (p=0.042) and compared to the iPD (2.05 ± 0.55) (p=0.044). Ratio of GCase protein over total actin expression as a loading control. ( **F**) Fibroblasts were stained for GCase (green), Lamp1 (red) and DAPI (blue). Representative images of Control and PD/GD lines. Density was measured in Image J. ( **E**) Western blot analysis was repeated in three independent experiments for each cell line. (A.U arbitrary units).*p<0.05. Data was found to be parametric and analysed by ANOVA and Bonferroni post hoc test. Analysis was repeated in three independent experiments for each cell line. Data are presented as mean ± s.e.m. ( **G**) Brightfield images of cell lines with AuNP uptake. No significant difference was found when measuring gold nanoparticle density in control (1.91 × 10 ^6^ ± 5.89 × 10 ^5^), PD/GD (1.31 × 10 ^6^ ± 1.79 × 10 ^5^), GD (1.24 × 10 ^6 ^± 3.41 × 10 ^5^), PD GBA (1.19 × 10 ^6 ^± 9.04 × 10 ^4^) and iPD (1.17 × 10 ^6 ^± 2.11 × 10 ^5^) (all p>0.05) (A.U) arbitrary units. Density was measured in Image J. For each cell line 30 fields of view were analysed from three separate repeats. The AuNP uptake data was parametric and analysed by ANOVA and Bonferroni post hoc test. Analysis was repeated in three independent experiments for each cell line. Data are presented as mean ± s.e.m. Scale bar = 100μm.

We started by assessing enzyme activity as an indication of overall lysosomal health and the extent to which this is restricted to GCase activity. A panel of lysosomal enzymes were screened for activity levels and included GCase, α-galactosidase, hexosaminidase and mannosidase. The enzyme activity was assessed in all the patient and healthy control fibroblast lines (n=17), and then grouped in to their respective disease phenotypes, based on GBA genotype for the analysis. GCase activity was found not surprisingly to be significantly lower in the PD GBA, GD and PD/GD compared to controls. In contrast, the iPD group was not significantly different compared to any other groups ( Figure 1A and Dataset 1). There was no significant difference seen in the other lysosomal enzymes that were looked at ( Figure 1 B–D) and Dataset 1).

Endogenous GCase protein expression was assessed by western blot to check if there were differences in the protein concentration between the lines (n=17). The PD/GD skin fibroblasts had significantly lower GCase protein compared to control, and iPD. There was no significant difference seen between GD, PD GBA and the HFL1 ( Figure 1E and Dataset 1). Fluorescence immunostaining revealed that the endogenous GCase and Lamp1 protein co-localised in the control lines but this was not the case in the PD/GD lines ( Figure 1F and Dataset 1).

We then measured lysosomal function by looking at the uptake of gold nanoparticles (AuNP), and did not find any significant difference when comparing all cell lines ( Figure 1G Dataset 1). Taken together, our result show a decrease in GCase levels and activity in GD and PD patients carrying GBA mutations, compared to iPD and healthy donors. However, the lysosomal basal function and content are not affected.

### Autophagy is affected in GD and PD GBA patient fibroblasts

Autophagy has a role in quality control in the cell, and to examine this we looked at autophagosome maturation as a measure of autophagic flux in the patient fibroblasts (n=17). We first investigated the autophagosome content by measuring the ratio of LC3IIb over total actin, in cells with or without Bafilomycin A1 treatment followed by starvation. Bafilomycin A1 is a specific inhibitor of vacuolar (H+)-ATPases, and a blocker of autophagosome-lysosome fusion. If the cell has a well-regulated autophagic flux, Bafilomycin A1 should increase LC3IIb without any starvation. If the cell has normal basal flux, starvation should increase LC3IIb, due to activation of autophagy. Using a combined starvation and Bafilomycin A1 treatment should produce the maximum amount of autophagosomes possible under these starvation conditions.

**Figure 2.**
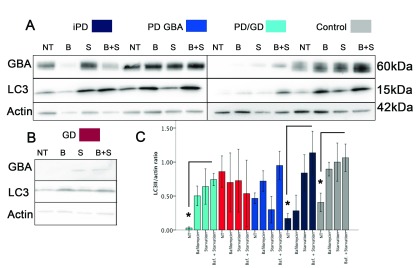
Autophagy assay in patient fibroblasts. ( **A, B**) Western blot of LC3IIb and GBA for all patient lines. Ratio of LC3 IIb/actin for control (NT) (0.41 ± .14) compared to (B+S) (1.06 ± 0.20) (p=0.053), iPD (NT) (0.17 ± .07) compared to (B+S) (1.13 ± 0.32) (p=0.032). This was also true for PD/GD (NT) (0.03 ± 0.01) compared to (B+S) (0.74 ± 0.09) (p=0.048), PD GBA (NT) (0.50 ± 0.07) compared to (B+S) (0.95 ± 0.21).GD (NT) (0.86 ± 0.23) compared to (B+S) (0.54 ± 0.49) (p>0.05). Optical density of western blot bands was measured in Image J. ( **C**) Data was analysed by ANOVA and Bonferroni post hoc test. Analysis was repeated in three independent experiments for each cell line. Data are presented as mean ± s.e.m.

In light of this, autophagic flux was found to be elevated following treatment with Bafilomycin A1 and starvation in control, iPD and PD/GD fibroblasts ( Figure 2 A–C and Dataset 2). In the GD and PD GBA lines, Bafilomycin A1 treatment had no effect on LC3IIb expression levels, indicating that autophagic flux was impaired in these cells ( Figure 2 and Dataset 2).

Raw data underlying the results presented in Figure 1 Figure 1A. GCase assay raw data and statistics.
Figure 1B. α-galactosidase assay raw data and statistics.
Figure 1C. Hexominidase assay raw data and statistics.
Figure 1D. Mannosidase assay raw data and statistics.
Figure 1E. Western blot GCase raw data, blots and statistics.
Figure 1F. Immunocytochemistry fibroblasts were stained for GCase (green), Lamp1 (red) and DAPI (blue). Representative images of control and PD/GD lines.
Figure 1G. Gold nanoparticles, images, raw data and statistics. Click here for additional data file. Copyright: © 2018 Collins LM et al. 2018 Data associated with the article are available under the terms of the Creative Commons Zero "No rights reserved" data waiver (CC0 1.0 Public domain dedication).

Raw data underlying the results presented in Figure 2 Figure 2A. Western blots for autophagy assay with iPD, PD GBA, PD/GD and control.
Figure 2B. Western blots for autophagy assay with GD.
Figure 2C. Autophagy assay statistics. Click here for additional data file. Copyright: © 2018 Collins LM et al. 2018 Data associated with the article are available under the terms of the Creative Commons Zero "No rights reserved" data waiver (CC0 1.0 Public domain dedication).

## Discussion

Our results show, for the first time in a large variety of PD/GD patient fibroblasts that the only lysosomal enzyme affected by GBA mutations is GCase. This finding is consistent with other’s work, who have shown GCase to be impaired in fibroblasts from GD and PD patients although in smaller patient numbers ^23– 26^. However we have now extended these findings to show that GCase is also abnormal in fibroblasts from patients with PD/GD and normal in iPD fibroblasts. We also found that the impairments are not due to low protein amounts in line with other studies ^24, 25^. This is in contrast with findings in brain post mortem tissue, where GCase amounts have been reported to be decreased in both PD GBA and iPD cases ^27^. In the case of PD/GD and GD the GCase protein and activity was reduced which was expected, however, in the PD GBA the protein amount was not reduced but the activity was reduced in this case, this leads us to believe in the case of PD GBA there is an additional factor, outside the GCase protein, causing pathology in these cells which could lead to dysfunction. In contrast to other studies, we did not find that the iPD line had significantly lower GCase activity compared to controls, although we were looking at fibroblasts rather than specific neuronal cells.

The screening of such a large number of GBA carriers with PD and GD, using a panel of lysosomal enzymes has not been previously undertaken. During this screening we found that there were no impairments in any other lysosomal enzymes measured besides GCase. These findings contradict published work which have reported that Hexosaminidase is elevated in PD GBA, GD and healthy GBA carriers fibroblasts ^24^. However, in this paper the enzyme activity was not directly measured, and the amount was determined by western blot, thus the protein amount may be elevated, but the specific activity may not be impaired.

In terms of looking at autophagic function, we used both Bafilomycin A1 and starvation treatments and observed a normal LC3IIb level in the PD/GD, iPD and control lines. However, in the case of the PD GBA and GD lines there was no increase in LC3IIb flux upon treatment. Most of the GD lines had N370S mutations and the PD GBA lines were carriers of N370S and E326K. Our results are in agreement with recent publications that found impaired autophagic flux was apparent in fibroblasts from a GD patient with homozygous L444P mutations ^28^ and in cases with compound heterozygous (N370S/L444P) mutations ^29^. However, other studies have found no impairment in autophagy, but these GD patients had Saposin C, not GBA, mutations ^30^.

All of the PD/GD lines in our study were compound heterozygous and two out of the three cases included L444P. These cases had normal autophagic flux, and this was the first study to assess autophagy in patients with both diseases. It may be that having both diseases together also impacts on autophagy, but autophagic function differs depending on the GBA mutation or cell type studied. There are mixed findings in relation to autophagy in PD depending on the model being tested. Autophagy has been found to be increased in fibroblasts from patients with LRRK2 mutations ^31^, but in cell lines it is decreased when α-synuclein is overexpressed ^32^. Autophagy as also been found to be impaired in iPS derived DA neurons from a PD patient with GBA mutations ^33, 34^. In our study, autophagic flux was normal in iPD patients but abnormal in the PD GBA lines. This could be further assessed by correcting the GBA mutation using CRISPR/Cas9 gene editing to see if the impairment in autophagy is dependent on GBA gene status. In addition it may be that GCase activity and autophagy are affected by mitochondrial function, which has been implicated in PD GBA previously ^35^, but as of yet, not extensively studied. Another factor to consider is that the affected GCase in GBA related PD, may be in the wrong location such as trapped in the ER, which has previously been observed to occur ^24^.

In summary, the GBA mutated patient fibroblast lines had impairments of 20–60% GCase activity compared to controls. These diseased cells had normal lysosomal function, with independent lysosomal uptake. However, PD GBA and GD lines display abnormal autophagy. Our results from these genetically relevant patient cell models provide evidence that GBA mutations could lead to impaired GCase and autophagic function, which may translate to CNS neurons and thus clinical expression and progression. If so, this cell model could be used in novel drug development.

## Data availability

The data referenced by this article are under copyright with the following copyright statement: Copyright: © 2018 Collins LM et al.

Data associated with the article are available under the terms of the Creative Commons Zero "No rights reserved" data waiver (CC0 1.0 Public domain dedication). 

**Dataset 1: Raw data underlying the results presented in Figure 1.**

Figure 1A. GCase assay raw data and statistics.

Figure 1B. α-galactosidase assay raw data and statistics.

Figure 1C. Hexominidase assay raw data and statistics.

Figure 1D. Mannosidase assay raw data and statistics.

Figure 1E. Western blot GCase raw data, blots and statistics.

Figure 1F. Immunocytochemistry fibroblasts were stained for GCase (green), Lamp1 (red) and DAPI (blue). Representative images of control and PD/GD lines.

Figure 1G. Gold nanoparticles, images, raw data and statistics.

DOI, 10.5256/f1000research.12090.d178244 ^36^

**Dataset 2: Raw data underlying the results presented in Figure 2.**

Figure 2A. Western blots for autophagy assay with iPD, PD GBA, PD/GD and control.

Figure 2B. Western blots for autophagy assay with GD.

Figure 2C. Autophagy assay statistics.

DOI, 10.5256/f1000research.12090.d178246 ^37^
