# Supplementary material for: Dermal fibroblasts from patients with Parkinson’s disease have normal GCase activity and autophagy compared to patients with PD and GBA mutations
Source: F1000Res. 2018 Feb 9;6:1751. Originally published 2017 Sep 26. [Version 2] doi: 10.12688/f1000research.12090.2 (PMC5820594; doi:10.12688/f1000research.12090.2)
Supplement: Raw data underlying the results presented in Figure 1 — Figure 1A. GCase assay raw data and statistics. Figure 1B. α-galactosidase assay raw data and statistics. Figure 1C. Hexominidase assay raw data and statistics. Figure 1D. Mannosidase assay raw data and statistics. Figure 1E. Western blot GCase raw data, blots and statistics. Figure 1F. Immunocytochemistry fibroblasts were stained for GCase (green), Lamp1 (red) and DAPI (blue). Representative images of control and PD/GD lines. Figure 1G. Gold nanoparticles, images, raw data and statistics. [file f1000research-6-14923-s0000.tgz › c5f6c2f0-f109-4824-a348-2faf1b4bba10_Figure_1.zip!/E/Blot/gel 1 actin 31.7.14.pdf]

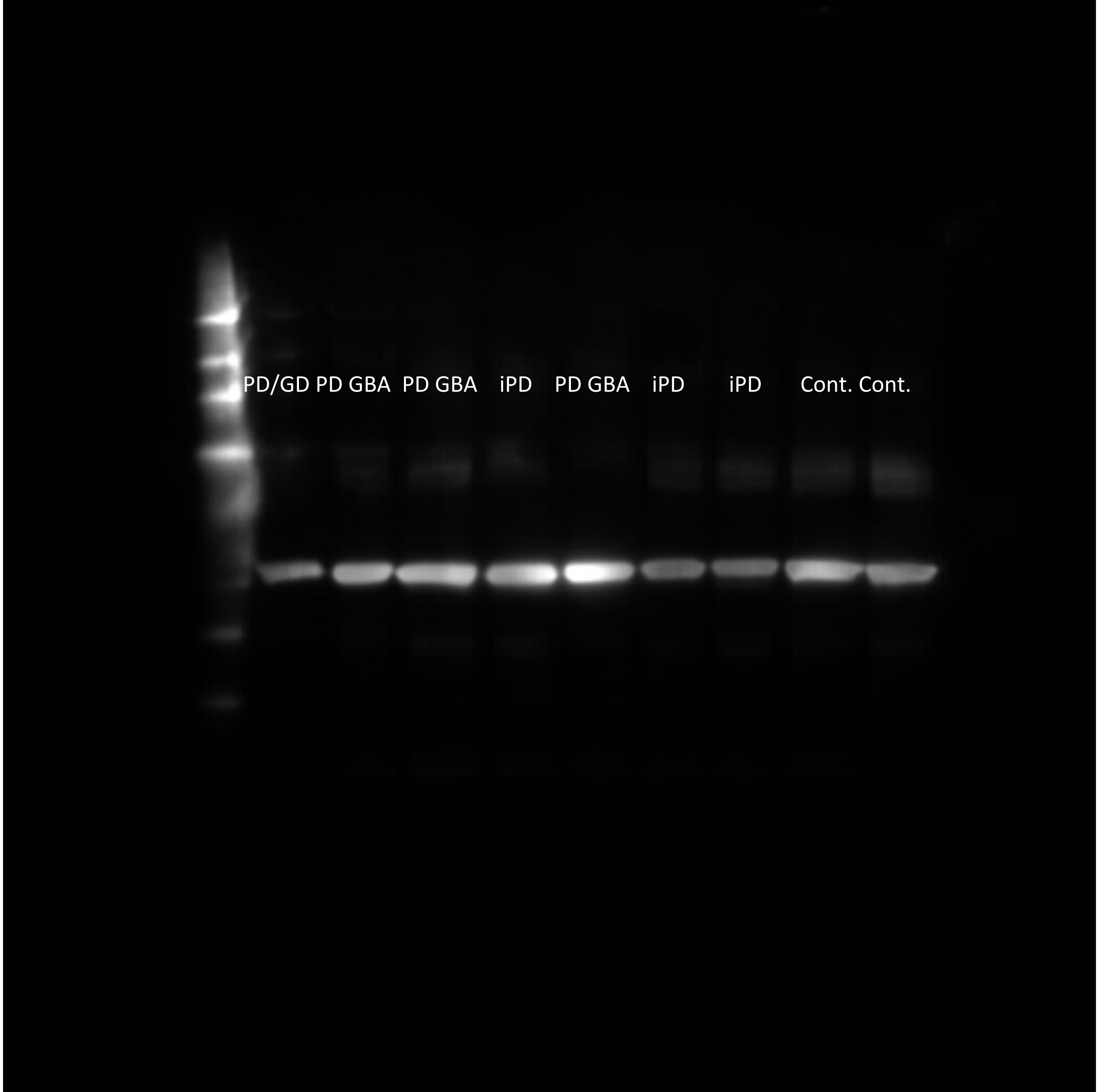
PD/GD
PD GBA
PD GBA
iPD
PD GBA
iPD
iPD
Cont.
Cont.
