# Supplementary material for: Dermal fibroblasts from patients with Parkinson’s disease have normal GCase activity and autophagy compared to patients with PD and GBA mutations
Source: F1000Res. 2018 Feb 9;6:1751. Originally published 2017 Sep 26. [Version 2] doi: 10.12688/f1000research.12090.2 (PMC5820594; doi:10.12688/f1000research.12090.2)
Supplement: Raw data underlying the results presented in Figure 2 — Figure 2A. Western blots for autophagy assay with iPD, PD GBA, PD/GD and control. Figure 2B. Western blots for autophagy assay with GD. Figure 2C. Autophagy assay statistics. [file f1000research-6-14923-s0001.tgz › 25f55834-88bf-4655-b82e-553001fc4ea3_Figure_2.zip!/B/GD actin blot.pdf]

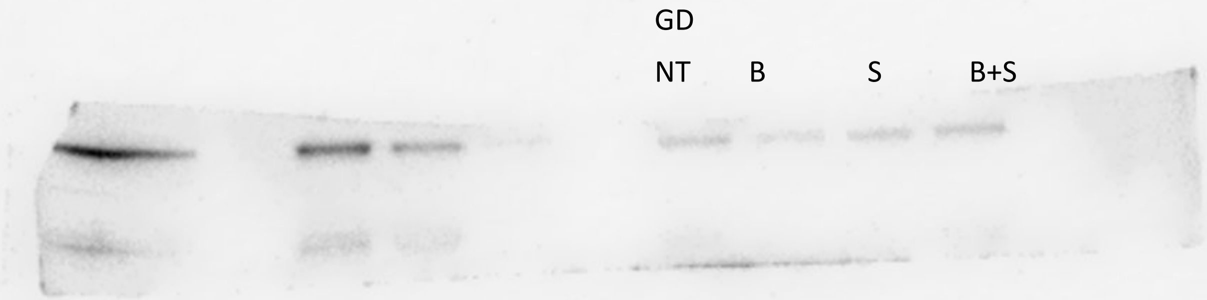
GD
NT
B
S
B+S
