# Supplementary material for: Dermal fibroblasts from patients with Parkinson’s disease have normal GCase activity and autophagy compared to patients with PD and GBA mutations
Source: F1000Res. 2018 Feb 9;6:1751. Originally published 2017 Sep 26. [Version 2] doi: 10.12688/f1000research.12090.2 (PMC5820594; doi:10.12688/f1000research.12090.2)
Supplement: Peer review history

# Peer review: Referee response for version 2

**DOI:** 10.5256/f1000research.14923.r30724

**Published:** 2018-02-16

**Authors:** Dario Besusso

**Affiliations:** 1 Laboratory of Stem Cell Biology and Pharmacology of Neurodegenerative Diseases, Department of Biosciences, University of Milan, Milan, Italy

I read through the new version and I am happy with the amendments.

I have read this submission. I believe that I have an appropriate level of expertise to confirm that it is of an acceptable scientific standard.

# Peer review: Referee response for version 1

**DOI:** 10.5256/f1000research.13081.r26388

**Published:** 2017-11-14

**Authors:** Mia Horowitz

**Affiliations:** 1 Department of Cell Research and Immunology, Tel Aviv University, Tel Aviv-Yafo, Israel

In the present manuscript, the authors used dermal fibroblasts to assess possible changes in acid- b-glucocerebrosidase (GCase) levels and changes in autophagy markers between three Gaucher disease (GD) patients with Parkinson disease, three carriers of GD mutations with PD, three idiopathic PD patients, four healthy controls and three GD patients with no known PD signs. They also had one human fetal lung fibroblast line (HFL1).

 The authors concluded that only PD patients with a GBA mutation have altered GCase activity and autophagy, which may explain their most rapid clinical progression. The authors mentioned that there was no decrease in GCase activity in iPD patients. However, the results in Figure 1A indicate reduced GCase activity in iPD patients. It may not be significant due to the small sample used in the study, but it is obvious and cannot be overlooked. Likewise, there is a notable decrease in the level of hexosaminidase and mannosidase activity. 

 The authors state that: "the impairments in (GCase) activity are not due to low protein amounts, in line with other studies". However, several reports have already documented decreased GCase amounts in GD derived cells. McNeill et al. (Reference 24 in the present manuscript) showed that: “Glucosylceramidase protein levels, assessed by western blot, were significantly reduced in fibroblasts from Gaucher disease (median glucosylceramidase levels 42% of control, P < 0.001) and heterozygous mutation carriers with (median 59% of control, P < 0.001) and without (median 68% of control, P < 0.001) Parkinson's disease". Other studies described a decrease in the amount of mutant GCase in cells that derived from GD patients and carriers of GD mutations. (Jonsson et al. ^1^; Ron et al. ^2^; Lu, et al. ^3^; Bendikov-Bar et al. ^4^).

 The authors mention in their discussion that: “Another factor to consider is that the affected GCase in GBA related PD **may be** in the wrong localization such as trapped in the ER”. It has already been shown that mutant GCase is recognized as misfolded in the ER, undergoes ER retention and, depending on the ability of the ER chaperones to refold it, it will either undergo ERAD or traffic to the lysosomes (Ron et al. ^2^). 

 Typographical errors:

1. The authors mention HFL1 or HLF cells and I guess they are the same.
2. GBA (or better GBA1, since there are three GBA genes: GBA1, GBA2 and GBA3) is the gene encoding acid-b-glucocerebrosidase (GCase). It is the protein (GCase) that localizes in the cell and not the GBA protein. It could also be referred to as the GBA1 encoded protein.
3. In Materials and Methods: bafilomycin assay: ….”this concentration was derived from titrations of Bafilomysin A1 **and 0.1mM and found** …” The second and should be replaced by was. In the same paragraph the authors mention that after NP-40 treatment “the cells were collected”. NP40 is a detergent that disrupts the plasma membranes so following its treatment there are lysates, not cells.
4. In the galactosidase assay the authors mention: “4-MUI  a-D-galactopyranosidase”, should be: 4-MU a-D-galactopyranoside.

I have read this submission. I believe that I have an appropriate level of expertise to confirm that it is of an acceptable scientific standard.

## References

[1] JonssonLMMurrayGJSorrellSHStrijlandAAertsJFGinnsEIBarrangerJATagerJMSchramAW: Biosynthesis and maturation of glucocerebrosidase in Gaucher fibroblasts. *Eur J Biochem*.1987;164(1) :171-9 354930110.1111/j.1432-1033.1987.tb11008.x

[2] RonIHorowitzM: ER retention and degradation as the molecular basis underlying Gaucher disease heterogeneity. *Hum Mol Genet*.2005;14(16) : 10.1093/hmg/ddi240 2387-98 10.1093/hmg/ddi240 16000318

[3] LuJChiangJIyerRRThompsonEKaneskiCRXuDSYangCChenMHodesRJLonserRRBradyROZhuangZ: Decreased glucocerebrosidase activity in Gaucher disease parallels quantitative enzyme loss due to abnormal interaction with TCP1 and c-Cbl. *Proc Natl Acad Sci U S A*.2010;107(50) : 10.1073/pnas.1014376107 21665-70 10.1073/pnas.1014376107 21098288PMC3003013

[4] Bendikov-BarIRonIFilocamoMHorowitzM: Characterization of the ERAD process of the L444P mutant glucocerebrosidase variant. *Blood Cells Mol Dis*.2011;46(1) : 10.1016/j.bcmd.2010.10.012 4-10 10.1016/j.bcmd.2010.10.012 21106416

# Response

**Published:** 2018-01-11

**Authors:** Lucy Collins

**Affiliations:** Cambridge University Hospitals NHS Foundation Trust, UK

I have made these changes, thank you

# Peer review: Referee response for version 1

**DOI:** 10.5256/f1000research.13081.r26387

**Published:** 2017-10-13

**Authors:** Dario Besusso

**Affiliations:** 1 Laboratory of Stem Cell Biology and Pharmacology of Neurodegenerative Diseases, Department of Biosciences, University of Milan, Milan, Italy

In the manuscript by Collins L.M. et al., the authors sought to investigate glucocerebrosidase (GCase) activity and autophagic flux in fibroblast derived from Parkinson’s (PD) and Gaucher disease patients (GD). GD is specifically caused by mutations in the GCase gene that drive defective enzymatic activity. Since GCase mutation is the most single commonest genetic risk factor in PD and is associated with poorer prognosis, the authors set to compare PD, GD and PD-GD patient-derived fibroblast for enzymatic activity and autophagic flux.

 The major point of concern is the physiological relevance of the cell models studied. Stating the obvious, since both PD and GD have neurological components, it would have been more appropriated to reprogram the fibroblasts in iPSCs and then convert these in the relevant cell type to assess GCase activity and autophagy. Unfortunately, this requires a good year of work.

 Interestingly, the authors show that mutations to the GCase gene affect both enzymatic activity (Figure 1A) and amount of protein in PD/GD (Figure 1E - Maybe also in other GBA context, but the statistical power is too low to discern this aspect). Since the first can be a consequence of the latter, as a mere suggestion, it would be of interest to investigate this aspect in more detail maybe looking at protein stability, although also this goes beyond the aim of the present manuscript.

 To this point, the authors state in the discussion about the GCase activity in PD/GD “We also found that the impairments are not due to low protein amounts in line with other studies”. As far as this reviewer understand, there is a significant difference between PD/GD and controls as indicated in Figure 1E. Authors may want to clarify this point.

 Major comments:

1. The most obscure point to this reviewer is the fact that PD/GD fibroblasts carrying homozygous mutation in DBA show reduced enzymatic activity but normal autophagic flux when PD GBA and GD (carrying similar mutations) have abnormal flux. Could the authors elaborate better their considerations to this point in the discussion?
2. The patient-derived cell lines are actually 13 and not 17 since 4 are derived from healthy donor. Please, amend the entire text on this regard.
3. In Figure 1A, multiple bands are visible in the WB. It is not clear which band is accounted for quantification. Please, highlight the quantified band in the figure, if possible.
4. Figure 1F will benefit from a colocalisation plot and quantification. It is not clear how many cells have been recorder for this phenotype.

 

 Minor comments:

1. The second column of Table 1 should specify “GBA genotype” in the title, for clarity.
2. Acronyms are first presented in the method session. Since many readers do not take the time to go through the methods, I kindly suggest repeating the acronyms in the main text.
3. Figure 2C is missing legend for the color-coding. Although, is the same as previous figure, a reminder would improve reading.
4. Legend of Figure 1F it may contain text that actually belong to the panel 1E.
5. In Figure 2A, please specify the subtype of LC3 in the legend.
6. The antibody used for LC3 WB usually recognizes both the LC3II and LC3IIb form of the protein. I wonder whether the authors have an explanation for not detecting the LC3II.

I have read this submission. I believe that I have an appropriate level of expertise to confirm that it is of an acceptable scientific standard, however I have significant reservations, as outlined above.

# Response

**Published:** 2018-01-11

**Authors:** Lucy Collins

**Affiliations:** Cambridge University Hospitals NHS Foundation Trust, UK

Reviewer’s comments:

*The major point of concern is the physiological relevance of the cell models studied. Stating the obvious, since both PD and GD have neurological components, it would have been more appropriated to reprogram the fibroblasts in iPSCs and then convert these in the relevant cell type to assess GCase activity and autophagy. Unfortunately, this requires a good year of work.*

 Thank you kindly for your comments. We really appreciate your review of our manuscript. As you point out it would have been more valuable as a module of neurodegeneration to convert these cells to iPS and neurons relevant to the disease. Janelle is now pursuing this through iN models with success in different starting from different cell types.

*Interestingly, the authors show that mutations to the GCase gene affect both enzymatic activity (Figure 1A) and amount of protein in PD/GD (Figure 1E - Maybe also in other GBA context, but the statistical power is too low to discern this aspect). Since the first can be a consequence of the latter, as a mere suggestion, it would be of interest to investigate this aspect in more detail maybe looking at protein stability, although also this goes beyond the aim of the present manuscript.*

 Thank you for the suggestion. Indeed it would have been really useful to assess the protein stability and we will consider this for future work, in the lab.

*To this point, the authors state in the discussion about the GCase activity in PD/GD “We also found that the impairments are not due to low protein amounts in line with other studies”. As far as this reviewer understand, there is a significant difference between PD/GD and controls as indicated in Figure 1E. Authors may want to clarify this point.*

 To clarify this point in the case of PD/GD and GD the GCase protein and activity was reduced which was expected, however, in the PD GBA the protein amount was not reduced but the activity was reduced in this case, this leads us to believe in the case of PD GBA there is an additional factor, outside the GCase protein, causing pathology in these cells which could lead to dysfunction. We have clarified this point in the text.

*Major comments:*

1. *The most obscure point to this reviewer is the fact that PD/GD fibroblasts carrying homozygous mutation in DBA show reduced enzymatic activity but normal autophagic flux when PD GBA and GD (carrying similar mutations) have abnormal flux. Could the authors elaborate better their considerations to this point in the discussion?*

 

 In the PD GBA and GD lines there was no increase in LC3II flux upon treatment, and this impairment could be due to the GBA mutations in these cases. Most of the GD lines had N370S mutations and the PD GBA lines were carriers of N370S and E326K. Autophagy may vary between different patients and indeed different GBA mutations/ combinations of GBA mutations. However, our results from the GD line are in agreement with a recent publication where it was found that impaired autophagic flux was apparent in fibroblasts from a GD patient with homozygous L444P mutations (de la Mata et al., 2015). Also in agreement with our study, it was shown that three GD fibroblasts lines with compound heterozygous N370S/L444P mutations had decreased autophagic flux (Lay et al., 2012). Other studies have 144 Lysosomal Function in Fibroblasts From Gaucher and Parkinson’s Disease found no impairment in autophagy (Pacheco et al., 2007; Tatti et al., 2012), but this was seen in GD patients with Saposin C mutations (Vaccaro et al., 2010).

 All of the PD/GD lines in our study were compound heterozygous and two out of the three cases included L444P. These cases had normal autophagic flux, and this was the first study to assess autophagy in patients with both diseases. It may be that having both diseases together also impacts on autophagy, but autophagic function differs depending on the GBA mutation or cell type studied. It is very rare to have both PD and GD so it would also be interesting to assess autophagic function in iN cells with a-syn in these cases. Indeed recently it has been shown that loss of GCase caused impairment in autophagy that was associated with PP2A (protein phosphatase 2A), further leading to a-syn accumulation, albeit in a neuroblastoma cell line (Du et al., 2015). It would be interesting to test the function of PP2A in future experiments in the patient fibroblasts.

  

1. *The patient-derived cell lines are actually 13 and not 17 since 4 are derived from healthy donor. Please, amend the entire text on this regard.*

 

 Text has been amended:

 Fibroblasts were derived from dermal skin biopsies. In Table 1 the characteristics of the 13 patient derived and 4 healthy control fibroblast cell lines are summarised.

 Autophagy has a role in quality control in the cell, and to examine this we looked at autophagosome maturation as a measure of autophagic flux in the patient and healthy control fibroblasts (n=17).

 The enzyme activity was assessed in all the patient and healthy control fibroblast lines (n=17), and then grouped

  

1. *In Figure 1A, multiple bands are visible in the WB. It is not clear which band is accounted for quantification. Please, highlight the quantified band in the figure, if possible.*

 

 The whole band was taken for the quantification as there are multiple forms of GBA depending on cellular location and trafficking. Similar western blots of GBA have been seen by others (McNeill et al., 2014).

  

1. *Figure 1F will benefit from a colocalisation plot and quantification. It is not clear how many cells have been recorder for this phenotype.*

 

 This is a good suggestion. However, the images were captured during my PhD study and I no longer have access to a microscope or image analysis to complete this. For this study the images were included as qualitative data to display the distribution of GCase. Others prior to this study have already shown that GCase is indeed sequestered in the ER. (McNeill et al., 2014). For this qualitative study 10 fields of view were taken for each fibroblast line in three separate wells.

**Minor comments:**

1. *The second column of Table 1 should specify “GBA genotype” in the title, for clarity.*

 

 This has been updated on page 23

  

1. *Acronyms are first presented in the method session. Since many readers do not take the time to go through the methods, I kindly suggest repeating the acronyms in the main text.*

 

 I have added main acronyms to the main text.

  

1. *Figure 2C is missing legend for the color-coding. Although, is the same as previous figure, a reminder would improve reading.*

 

 This has been updated and included.

  

1. *Legend of Figure 1F it may contain text that actually belong to the panel 1E.*

 

 The text has been updated to correct this.

  

1. *In Figure 2A, please specify the subtype of LC3 in the legend.*

 

 The subtype used was LC3IIb, this has been added to the legend

  

1. *The antibody used for LC3 WB usually recognizes both the LC3II and LC3IIb form of the protein. I wonder whether the authors have an explanation for not detecting the LC3II*.

 

 The LC3II band was quite faint in these experiments. This could be due to the pathological affect of GBA in the disease states on the lysosome, as in the control lines the bands were more detectable. This could also be due to experimental technique as part of the bafilomycin assay requires washing and harvesting the cells and LC3 quite a small protein. It is possible that LC3 levels in the cell lines are very low, on the limit of detection. Also in these patient and healthy control lines there could be a rapid turnover of LC3.
